# Network pharmacology and experimental investigation of *Rhizoma polygonati* extract targeted kinase with herbzyme activity for potent drug delivery

**DOI:** 10.1080/10717544.2021.1977422

**Published:** 2021-10-18

**Authors:** Yingqiu Xie, Chengling Mu, Bexultan Kazybay, Qinglei Sun, Aidana Kutzhanova, Guldan Nazarbek, Na Xu, Lazzat Nurtay, Qian Wang, Amr Amin, Xugang Li

**Affiliations:** a Department of Biology, School of Sciences and Humanities, Nazarbayev University, Nur-Sultan, Kazakhstan; b Sino-German Joint Research Center on Agricultural Biology, State Key Laboratory of Crop Biology, College of Life Sciences, Shandong Agricultural University, Tai'an, Shandong Province, China; c Key Laboratory for Applied Technology of Sophisticated Analytical Instrument of Shandong Province, Shandong Analysis and Test Center, Qilu University of Technology (Shandong Academy of Sciences), Jinan, China; d Shandong Taishanghuangjing Biotechnology Co. Ltd., Tai'an, China; e Biology Department, UAE University, Al Ain, United Arab Emirates; f The College, The University of Chicago, Chicago, IL, USA

**Keywords:** Huangjing, herbzyme, traditional Chinese medicine

## Abstract

*Rhizoma polygonati* (Huangjing, RP) has been used for a long history with many chemical components in inducing anti-cancer, anti-aging, anti-diabetes, anti-fatigue, and more prevention of diseases or acts as nutrition sources in food. Here we investigated RP extract combination with kinase inhibitors in anti-cell growth and blockade in pathways targeting kinases. Experimental investigation and network pharmacology analysis were applied to test the potent kinase-mediated signaling. Herbzyme activity was determined by substrate with optical density measurement. Extract of processed RP inhibits cell growth in a much greater manner than alone when applied in combination with inhibitors of mTOR or EGFR. Moreover, processing methods of RP from Mount Tai (RP-Mount Tai) play essential roles in herbzyme activity of phosphatase suggesting the interface is also essential, in addition to the chemical component. The network pharmacology analysis showed the chemical component and target networks involving AKT and mTOR, which is consistent with experimental validation. Finally, EGFR inhibitor could be associated with nano-extract of RP-Mount Tai but not significantly affects the phosphatase herbzyme activity *in vitro*. Thus the processed extract of RP-Mount Tai may play a dual role in the inhibition of cell proliferation signaling by both chemical component and nanoscale herbzyme of phosphatase activity to inhibit kinases including mTOR/AKT in potent drug delivery of kinase inhibitors.

## Introduction

*Rhizoma polygonati* (Huangjing, RP) is a ‘medicine food homology’ (MFH) known to be used in Traditional Chinese Medicine (TCM) for thousands of years with many natural products to maintain proper blood sugar levels, and exhibit many more pharmacological functions (Zhao et al., 2018, 2021). Recently it has been shown that RP can also be used for the prevention of COVID-19 (Mu et al., 2020, 2021). RP components include polysaccharides, alkaloids, lectins, saponins (Zhao et al., 2018). Traditionally, this herb was used to make medicine against obesity, fatigue, high temperature, and common cold (Zhao et al., 2018). Modern pharmacology also provides evidence of the efficacy of RP’s chemical components against diabetes, aging, Alzheimer’s disease, cardiovascular diseases, and viral infection (Zhao et al., 2021).

Recent research outcomes illustrate that RP’s ingredients could also be used against kinases including p38MAPK (Han et al., 2020), glycogen synthase kinase 3β (GSK-3β), extracellular signal-regulated kinase (ERK) (Peng et al., 2018). Consistently, we reported the potent herbzyme phosphatase-like herbzyme activity inhibiting cell growth, simulating the kinase targeting by RP herbzyme function (Benassi et al., 2021). Thus RP herbzyme was hypothesized to be linked to the crosstalk between kinase and phosphatase signaling pathways in cell growth.

It is well-documented that kinases play essential roles in the regulation of metabolic processes of cells and are further involved in critical differentiation, proliferation, apoptosis, and cell development (Fabbro et al., 2015). Studies also show that phosphatases are involved in these phosphorylation–dephosphorylation processes along with kinases. It has been recognized that phosphatase might be essential in targeting cancer cell growth by disrupting kinase signaling pathways (Heinrich et al., 2018). Therefore, we hypothesize that nanozymatic phosphatase activity of RP might inhibit or disrupt the kinase signaling pathways, hence blocking cellular proliferation including cancer cells. It is additionally expected that RP ingredients could have a larger effect in combination with precision kinase inhibitor drugs against cancer cell growth. We further examined whether RP ingredients could have the potential to be used to inhibit cell growth in combination with kinase inhibitors which might be applied in treatments against other types of diseases in the future as well because many diseases are induced or related to kinase signaling for their progression.

Network pharmacology provided computational and systems tools to examine and explain the mechanisms underlying the effect of drugs by constructing compound-based target protein-protein interactions following the molecular modeling technology with calculations of stoichiometry to simulate compound ligand-target binding (Jiao et al., 2021). Here, both mentioned methods were used to identify and understand how RP extract can target cell growth by inhibiting kinases in potent drug delivery of kinase inhibitors.

## Materials and methods

### Cell line and cell viability analysis

HT-1080 cells were used for cell-based studies. HT-1080 cells are human cells originally derived from a 35-year-old White male patient with a disease of fibrosarcoma (ATCC, CCL-121, https://www.atcc.org/products/ccl-121). Cells were plated in 24 well plates with a density of 5000 cells/well for overnight followed by treatment with vehicle or agent and after 3–4 days cells were fixed and stained with crystal violet. Finally, cells were photographed and measured by optical density at 590 nm. The agents used for inhibition of differential signaling are olaparib (PARP inhibitor (i), PARPi (Sigma Aldrich), crizotinib (MET kinase inhibitor, METi, Sigma Aldrich), staurosporine (PKCi), SMI-4a (CAS 327033-36-3, Pim-1/2 inhibitor Pim-1/2i, Pimi, Santa Cruz), erlotinib Hydrochloride (EGFRi, Santa Cruz), rapamycin (mTORi), iCRT3 (β-catenin inhibitor) and verteporfin (CAS 129497-78-5, YAPi, Santa Cruz).

### Collection of kinase-related gene sets

The genes of targets of kinases were retrieved from the GeneCards (https://www.genecards.org/) database, NCBI (https://www.ncbi.nlm.nih.gov) gene database, and GenCLiP3 (http://ci.smu.edu.cn/genclip3/GeneAssociation.php) database using ‘kinase’ as the keyword while searching and selecting the species as ‘Homo sapiens’ to obtain kinase-related human genes. Venny 2.1.0 (https://bioinfogp.cnb.csic.es/tools/venny/) software (Sun et al., 2019) was used to identify the targets of RP compounds against the kinase-related genes.

### Construction of protein-protein interaction (PPI) network and compound-target-pathway network

The potential targets were used to construct the target PPI network through the STRING database (Szklarczyk et al., 2019) (Search Tool for the Retrieval of Interacting Genes/Proteins, https://string-db.org/) where the ‘Homo sapiens’ keyword was applied before running the Multiple Proteins service of the STRING. Further, Cytoscape 3.7.2 (Shannon et al., 2003) software (http://www.cytoscape.org/) was used to construct a protein interaction network and identify the most important RP’s protein targets for molecular docking analysis.

### Network pharmacology analysis of target GO enrichment analysis and KEGG pathway enrichment analysis

To further understand the functions of core target genes and the main action pathways of the active substances of RP, the disease-related targets obtained from the above screening were entered into the DAVID database (Huang et al., 2009) (https://david.ncifcrf.gov/home.jsp). The list of genes of RP targets against kinases was uploaded to the DAVID searching tool. Here, the species section was selected as ‘homo sapiens’, and all target gene names were corrected to their official names (official gene symbol), and the threshold of *p* < 0.05 was set for GO enrichment before running the tool.

For KEGG enrichment pathway analysis the core target genes were imported into the KOBAS3.0 database (http://kobas.cbi.pku.edu.cn). Using *p* < 0.05 and sorting from small to large as the screening conditions, the enrichment analysis pathways were selected which then were visualized through online tool (http://www.bioinformatics.com.cn).^1^

### Virtual screening of the active ingredients of RP and online tools

The active ingredients of RP were obtained by searching the Traditional Chinese Medicine System Pharmacology database (TCMSP) (http://tcmspw.com/index.php) and the BenCaoZuJian database (http://herb.ac.cn/) (Fang et al., 2021). To confirm there is of RP ingredients the TCMSP results were checked by the Swiss Target Prediction (http://www.swisstargetprediction.ch/) database. Additionally, the bioinformatics online mapping website (http://www.bioinformatics.com.cn)^1^ was used to visualize the analysis results.

### Molecular modeling of RP compounds binding AKT1 and mTOR

The 3 D structure of the effective active RP compounds was retrieved through the PubChem website (https://pubchem.ncbi.nlm.nih.gov/) in the SDF file format which was then converted into a PDB file using the Open Babel 2.3.2 software. The receptor protein was retrieved from the Protein Data Bank database (http://www.rcsb.org/pdb). For AKT1 docking, the PDB structure of 4EKL was used which is bound with GDC0068 by amino acids of Phe 22, Lys 40, Ala91, Glu95, Asp 153 (https://www.rcsb.org/sequence/4EKL). GDC-0068 is a highly selective pan-AKT inhibitor. The PYMOL software was further used to perform operations, such as water removal and ligand removal on the receptor protein, while the Autodock 4.2.6, and AutoDock Vina 1.1.2 (Trott & Olson, 2010) were used to set the parameters of the receptor protein docking site including active pocket sites for molecular docking of receptor proteins and small ligand molecules after the molecules were converted into pdbqt format. For mTOR structure, PDB 4JSV was used as a reference structure.

### Herbzyme phosphatase assay

The herb enzyme of phosphatase assay was tested with substrate NBT/BCIP (Nitro blue tetrazolium/5-bromo-4-chloro-3-indolyl phosphate) and absorbance measurement (Benassi et al., 2021). The Calf-intestinal alkaline phosphatase (CIP, Life Technologies) was used as a positive control and vehicle water as a negative control for RP.

### Transmission electron microscopy (TEM) and scanning electron (SEM) microscopy

TEM test was performed by the University core facility with the equipment of Transmission electron microscope JEOL JE-1400 plus. Samples were applied on a carbon grid (diameter: 3 mm) and dehydrated for at least 24 h. SEM investigation was operated by loading RP extract on papers of foil and air-dry before putting it into the machine. Finally, the samples were investigated by Zeiss SEM microscope.

## Results and discussion

### Different processing generated RP Mount-Tai extract with nanoscale herbzymatic phosphatase activity

**Figure 1.**
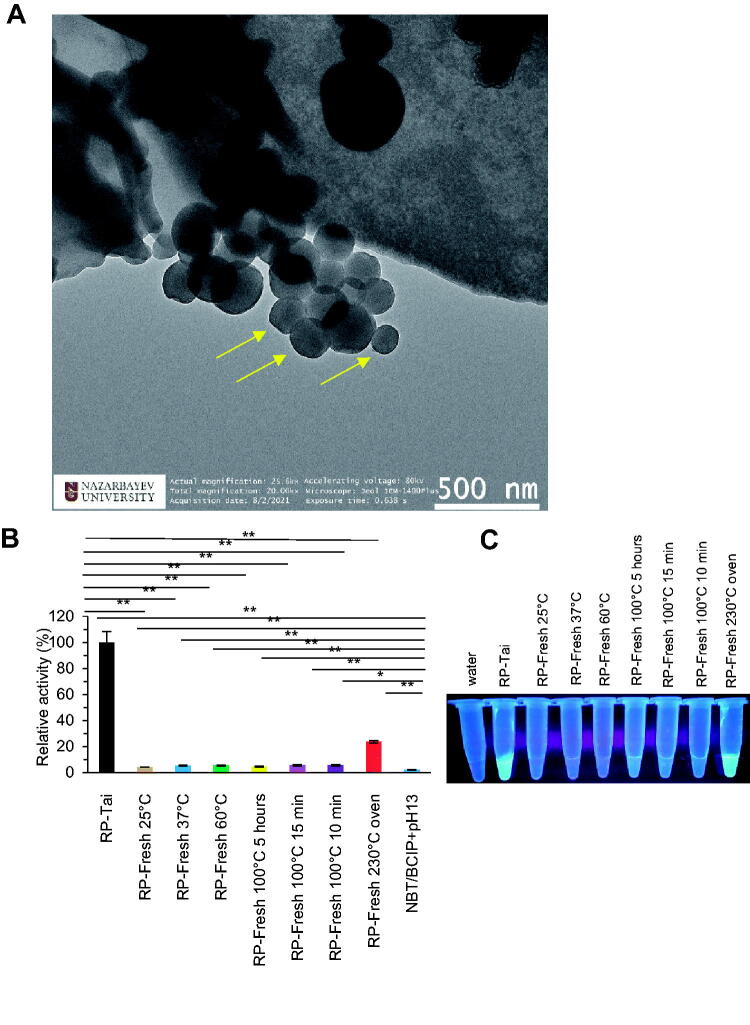
RP extract exhibits phosphatase activity by processing at high temperatures related to the nanoparticle effect. (A) TEM of processed RP extract at the nanoscale. (B,C) RP extract exhibits phosphatase activity by processing at high temperature measured by substrate NBT/BCIP (B) and correlates with quantum characteristics with fluorescence (C). *p<0.05; **p<0.01.

An ancient way of RP processing includes many times steam and sun-drying, which is unique to RP, compared to most herbs processing like boiling, or fresh use. We applied for a RP from a mountain area of Mount-Tai, China for further studies. We first reproducibly found the characteristics of ancient ways of processing generated nanoparticles in RP extract. We found the density plays an essential role in nanoparticle accumulation and high density may produce cluster simulating polymers of the nanostructures investigated by SEM (Benassi et al., 2021) and TEM (Figure 1(A)). Next, we found the nanoparticles of RP extract by commercial processing showed the highest activity of phosphatase compared to any other processed particles. RP baked at a high temperature of 230 °C can also generate enzyme activity, named by us, herbzyme, and a natural type of nanozyme (Figure 1(B)). The fluorescence investigation showed the high temperature of 230 °C processed RP extract has high fluorescence with high phosphatase activity (Figure 1(C)). The correlation of fluorescence with phosphatase activity in anciently processed RP and 230 °C processed RP suggesting the characteristics of the nanoparticles correlate with the nanozyme/herbzyme activity of phosphatase. Compared to the metal-based nanozyme, this natural processed herbal nanozyme seems cost-effective and easily absorbs with potential in drug delivery (Meng et al., 2021).

**Figure 2.**
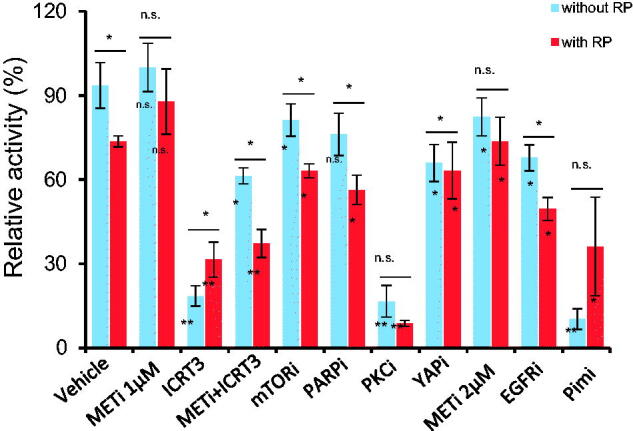
RP extract inhibits cell growth enhanced by combination with kinase inhibitors including mTOR in HT-1080 cells. Cells were treated by RP extract (5 mg/mL), Olaparib [PARP inhibitor (i), PARPi at 5 µM], crizotinib (METi, 1 µM or 2 µM), staurosporine (PKCi, 0.17 µM), SMI-4a (Pim-1i or Pim-2i, 20 µM), Erlotinib Hydrochloride (EGFRi,20 µM), rapamycin (mTORi, 0.1 µM), iCRT3 (β-catenin inhibitor, 30 µM) and Verteporfin (YAPi, 0.1 µM) alone or combination as indicated. The significance between with RP or without RP was indicated between blue bar *vs.* red bar. *p <0 .05; **p <0 .01. For controls of vehicle or RP alone, each inhibitor effect was compared and indicated to the two controls inside the bar of blue or red, respectively.

Given phosphatase plays a role in removing phosphorylation of target against kinases, we tested the relation between RP extract phosphatase function and kinase inhibitors in combination to inhibit cell growth in HT-1080 cells. We found that MET, PKC, Pim-1/2 kinases inhibitors did not show the synergistic association in inhibition of cell growth, while mTOR and EGFR kinases inhibitors showed the effect (Figure 2). Thus the RP extract mediated phosphatase activity may crosstalk with mTOR, EGFR related pathways for kinase inhibition. The downstream related targets include YAP, and a potent inhibitor of β-catenin, named as iCRT3, with MET kinase inhibitor/RP combination showed the significantly decreased cell growth compared to other target inhibition results, such as YAP (Figure 2).

### Network pharmacology analysis of RP induced cell signaling against kinases including mTOR/AKT pathway

**Figure 3.**
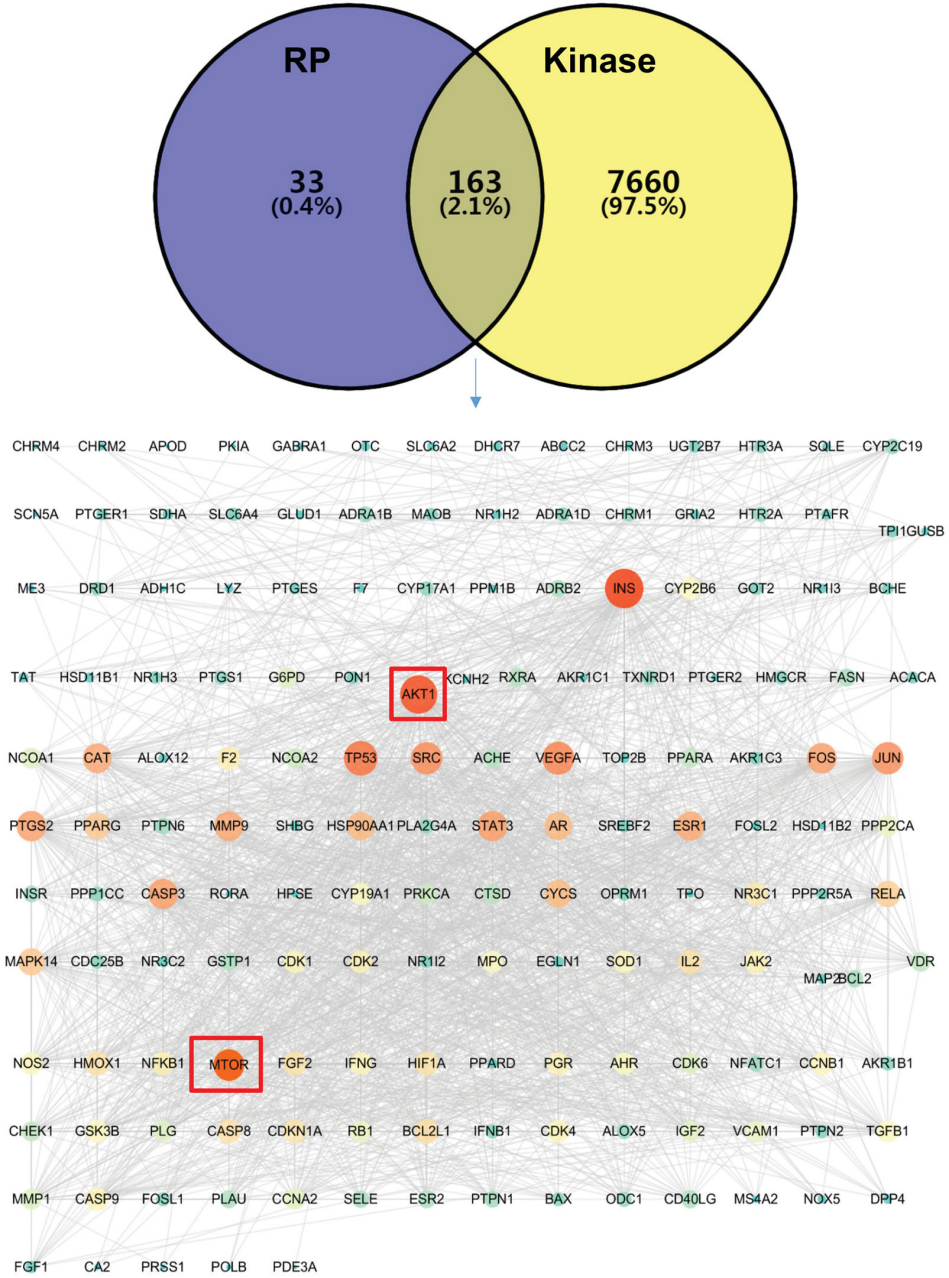
The intersection of RP compounds with corresponding targets and kinase-related genes. (A) Venn diagram of the targets corresponding to the RP compounds (purple circle) and the kinase-related gene set (yellow circle). The middle gray is the intersection target of the two. (B) The network of the common targets of RP compounds-kinase where nodes represent protein targets, while edges indicate their interactions. AKT1 and mTOR are of the higher degree of importance in this PPI network.

Next, we explored the whole RP chemical compound-targets correlations against kinases by network analysis. With the Venn diagram tools, we searched the intersection targets of kinase signaling and RP target pathways and we found a total of 163 (2.1%) targets (Figure 3(A)). Then we constructed and analyzed the protein-protein interaction networks (Figure 3(B)) of these intersection targets via the STRING platform and Cytoscape 3.7.2 software, respectively. The PPI network contains a total of 162 nodes and 1985 edges. The nodes in the network graph represent proteins. The degree value represents the number of lines connected to the same node, which is used to evaluate the importance of each node in the network. The larger the node and the darker the color, the greater the degree value. Each edge represents the interaction relationship between proteins, and the more lines, the greater the degree of association. It can be seen from Figure 3(B) that the degree values of INS, AKT1, TP53, PTGS2, CAT, VEGFA, JUN, SRC, PPARG, MMP9, HSP90AA1, STAT3, ESP1, CASP3, MTOR, and FOS are significantly higher than those of other targets, implying that they play an important role in the PPI network. Among them, AKT1, INS, and MTOR have the largest nodes and the darkest colors, which may be the most important potential targets for their effects. Because the AKT1 and MTOR are major players of PI3K-AKT signaling pathway that have many functions including cell cycle progression, cell survival, and apoptosis, and also were regulated by RP compound polysaccharides in anti-neurotoxicity (Afify et al., 2021; Huang et al., 2021).

**Figure 4.**
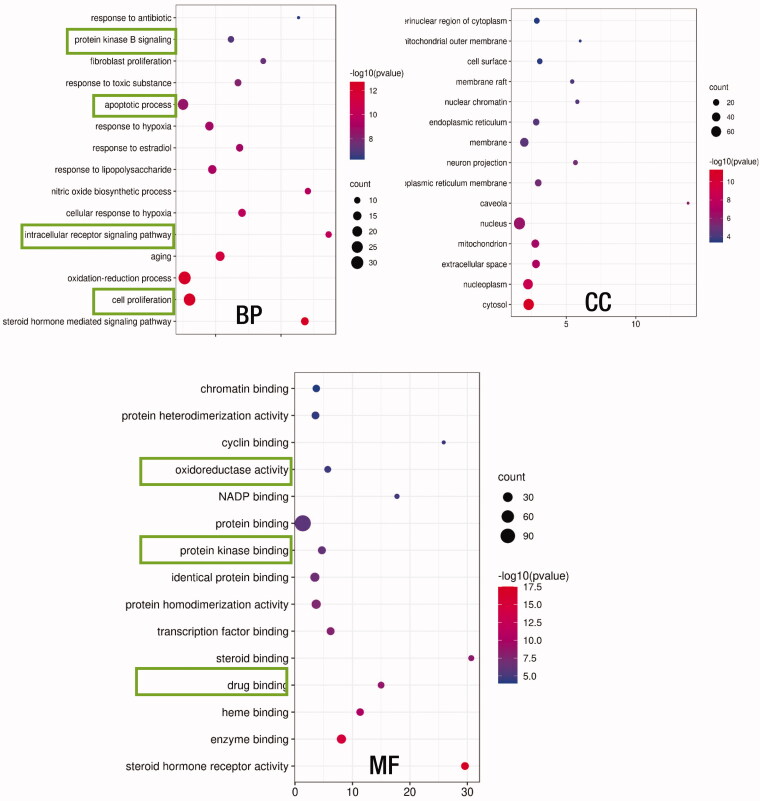
GO enrichment analysis of the RP compounds’ intersection targets. The intersection targets are submitted into the DAVID database for GO enrichment analysis. The figure shows biological processes (BP), cell components (CC), and molecular functions (MF). The size of the bubble in the figure represents the number of genes enriched in this entry, and the color of the bubble is expressed as − log10 (*p*-value). The larger the value, the redder the color, and the smaller the value, the bluer the color.

The targets corresponding to the RP compounds obtained from the TCMSP website were uploaded into the DAVID database, and 312 GO entries were screened out according to *p* ≤ .05. Among them, there are 228 biological processes (BP), accounting for 73%; 30 cellular components (CC), accounting for 9%; and 54 molecular functions (MF), accounting for 17%. The important biological processes were recorded as a response to an antibiotic, protein kinase B signaling, fibroblast proliferation, response to a toxic substance, apoptotic process, response to hypoxia, response to estradiol, response to lipopolysaccharide, nitric oxide biosynthetic process, cellular response to hypoxia, intracellular receptor signaling pathway, aging, oxidation-reduction process, cell proliferation, and steroid hormone-mediated signaling pathway (Figure 4). And the potential targets of RP compounds showed enrichment for MFs, such as chromatin binding, protein heterodimerization activity, cyclin binding, oxidoreductase activity, NADP binding, protein binding, protein kinase binding, identical protein binding, protein homodimerization activity, transportation factor binding, steroid binding, drug binding, heme binding, enzyme binding, steroid hormone receptor activity (Figure 4). From here, it can be claimed that the mechanism of the RP compounds involved in the kinase signaling may be related to the activity of steroid hormone receptor activity, oxidoreductase activity, cyclin binding activity, and other protein activities. At the same time, involved cellular components include a vast array of cellular structures starting from the extracellular space, membrane, and cytosol to the nucleus, chromatins, mitochondria, and endoplasmic reticulum drawing the path of kinase signaling cascade from outside of the cell to the DNA (Figure 4). Such results might imply that the RP compounds affect the kinase signaling at many cascade points illustrating their great potential to disturb and target the kinases involved, especially, the cellular growth and proliferation.

**Figure 5.**
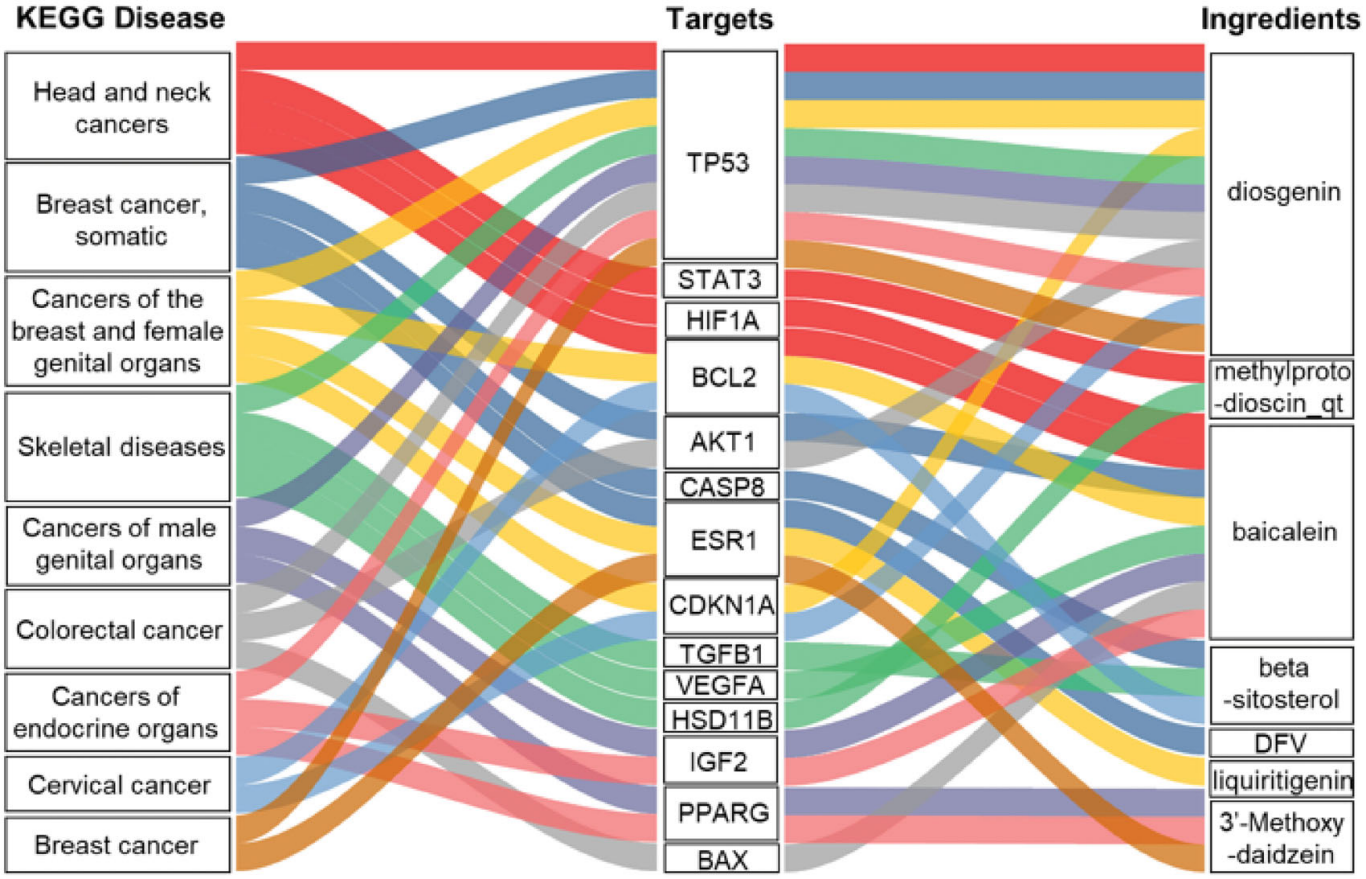
KEGG enrichment pathway analysis of the potential targets of RP compounds against kinases involved in cancer signaling. The diagram shows the KEGG diseases and the RP compounds corresponding to the intersection targets establishing the relationship between the compound and the various cancer types.

Further, the key common targets of the RP compounds and different KEGG diseases and the RP ingredients were input into the KOBAS 3.0 database, and a total of 133 diseases were identified. We used *p* ≤ 0.05 to screen 57 diseases and selected the top nine pathways for display (Figure 5) including head and neck cancers, somatic breast cancer, cancers of the breast and female genital organs, skeletal diseases, cancers of male genital organs, colorectal cancer, cancers of endocrine organs, cervical cancer, and breast cancer. These records illustrate the RP compounds mainly target cancer compared to other diseases, which might be due to the kinases that are important for cancer cell growth. Moreover, the constructed disease-compound-target relationship diagram below also shows that the main ingredients are diosgenin, methylprotodioscin, baicalein, beta-sitosterol, DFV, liquiritigenin, and 3′-Methoxydaidzein. While diosgenin could target all nine KEGG diseases and baicalein 7 of them, others seem to have the potential to target more specific diseases. These top seven RP ingredients were further investigated via molecular docking.

**Figure 6.**
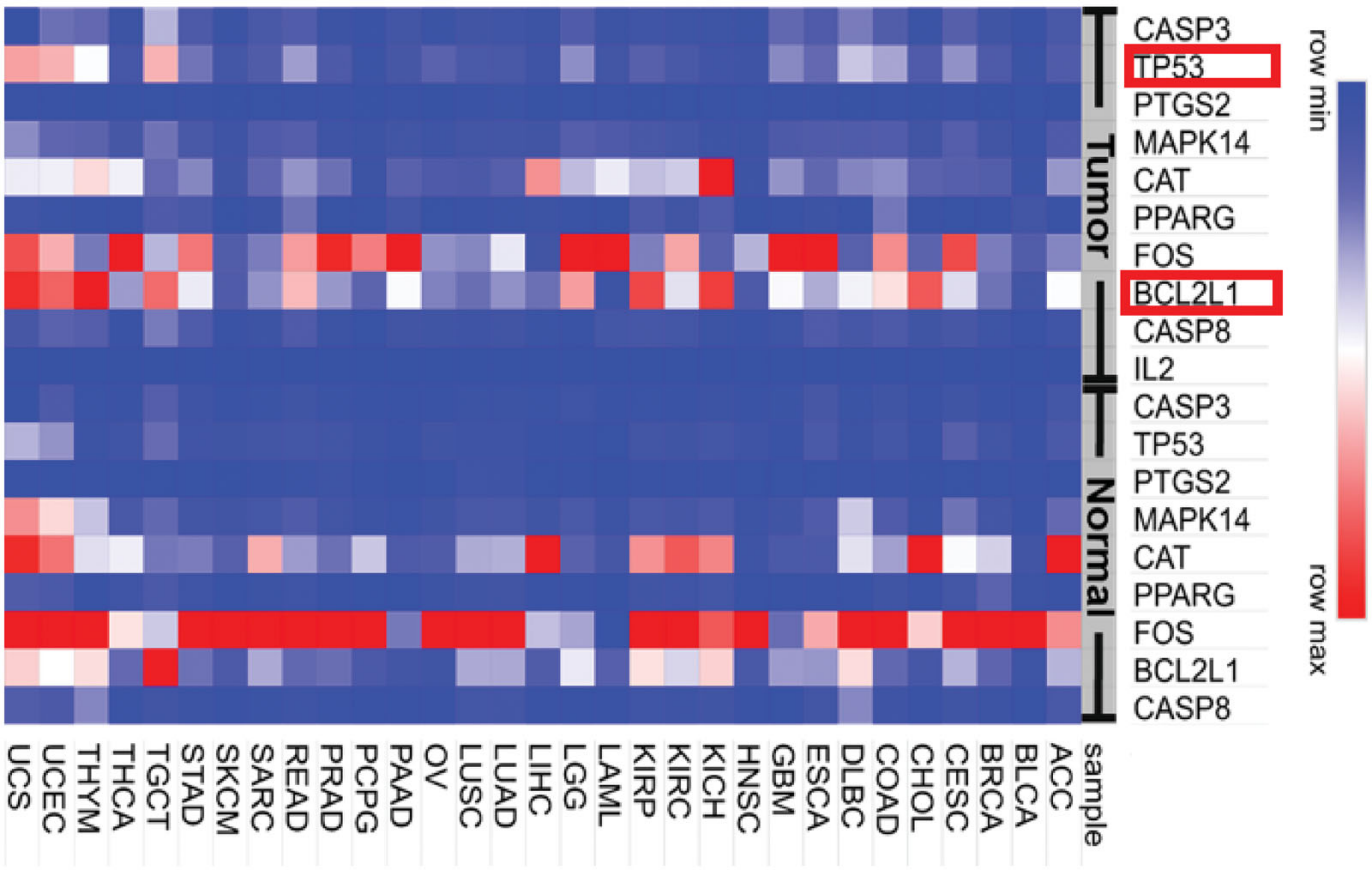
Gene expression of RP target genes in different cancer types. The potential targets of RP compounds were uploaded to the GEPIA website to obtain gene expression under normal (control group) conditions and cancer (Tumor) conditions. The gene expression level is indicated on the right side with blue being low expression (minimal) and red being high expression (maximum).

For further analysis, the GEPIA website was used. The gene list of potential targets of RP compounds was uploaded into this service which generated the gene expression map of nine genes (CASP8, BCL2L1, FOS, PPARG, CAT, MAPK14, PTGS2, TP53, and CASP3) against 31 sample genes (Figure 6). There, while FOS, CAT, and MAPK14 were downregulated in the tumor, the BCL2L1 and TP53 were upregulated. The proteins of the latter two genes are involved in the PI3K-AKT-mTOR pathway. And because BCL2L1 and TP53 are responsible for cellular apoptosis, their upregulation in tumors means that the RP compounds can cause cancer cell death and consequently inhibit their proliferation.

### Molecular docking of the screened components of Rhizoma polygonati with AKT1 and mTOR

Molecular docking technology would allow to find the best binding pattern between molecules through spatial matching and energy recognition. Usually, the spatial matching of ligand and receptor binding is the basis for its function, and energy recognition is the key to maintain relatively stable binding. In general, the Vina system docking score value of less than certain energy (calculated by kcal/mol) indicates the binding between the docking molecule and the target (http://autodock.scripps.edu/faqs-help/manual/autodock-4-2-user-guide/AutoDock4.2_UserGuide.pdf) (Morris et al., 2009; Quiroga & Villarreal, 2016).

For the molecular docking, we used the AKT1 and mTOR protein targets which were identified to have a critical role as demonstrated by the network pharmacology analyses and seven RP ingredients identified to target main KEGG pathways. The results demonstrated that both AKT1 and mTOR have a strong binding affinity with RP ingredient molecules which were chosen for the molecular docking from the KEGG analysis as indicated by their docking scores, except for mannose, though it also showed relatively good binding affinity (about −5 kcal/mol). Also, similar to AKT inhibitor LY294002, the RP’s compound diosgenin has the strongest binding affinities with AKT1 (−8.7, −8.8 kcal/mol, respectively). In addition, similar to mTOR inhibitor Rapamycin, diosgenin also showed strong binding to mTOR (−10.4, −9.8 kcal/mol, respectively). See Table 1 for details.

**Table 1.** Molecular docking scores of AKT1 and mTOR with RP compounds*.

Molecule name	Docking score (kcal/mol)	
	AKT1	mTOR
Diosgenin	−8.8	−9.8
Sitosterol	−7.6	−8.5
Baicalein	−8.1	−8.6
DFV	−8.5	−8.2
Mannose	−5.3	−5.5
Positive control	−8.7** LY294002	−10.4*** Rapamycin

*The docking scores of AKT1 and mTOR against six molecules are provided in kcal/mol. Controls: **LY294002; ***Rapamycin.

**Figure 7.**
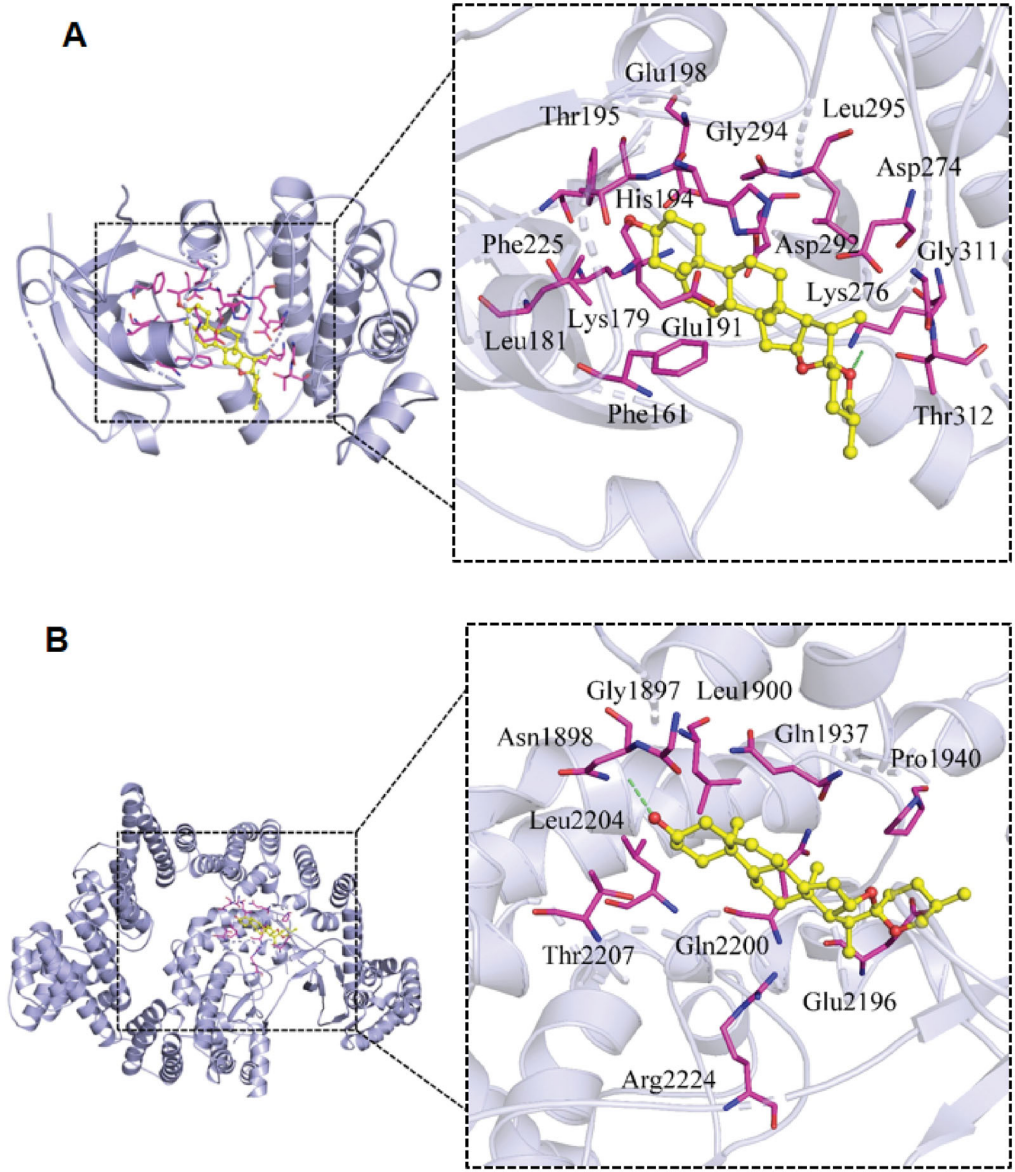
Molecular docking of diosgenin with the targets of RP compounds, AKT1, and mTOR. The binding mode between the small diosgenin ligand molecule and the receptor protein AKT1 (A) and the receptor protein mTOR (B). In both pictures, the 3 D docking model (left) is enlarged in the binding pocket site (right side within the dashed box). The AKT1 and mTOR are depicted in gray with their amino acids which are interacting in purple.

The molecular docking performed with diosgenin with AKT1 and mTOR target proteins displays that the binding pockets of both AKT1 and mTOR receptor proteins have many amino acid residues that form various bonds with diosgenin (Figure 7). Although the size of AKT1 is much smaller than mTOR, they have a similar number of residues that interact with diosgenin. AKT1 has Phe-161, Leu-181, Lys-179, Glu-191, His-194, Thr-195, Glu-198, Phe-225, Gly-294, Leu-295, Asp-274, Lys-276, Asp-292, Gly-311, and Thr-319, while mTOR has been recorded with Gly-1897, Asn-1898, Leu-1900, Glu-1937, Pro-1940, Glu-2196, Gln-2200, Thr-2207, Arg-2224, and Leu-2207. It can be observed that there are hydrogen bonds near Lys-276 of AKT1 (Figure 7(A)) and Asn-1898 in mTOR (Figure 7(B)), however, it is not clear which residues are involved. Thus, the binding pockets of both proteins were further studied.

**Figure 8.**
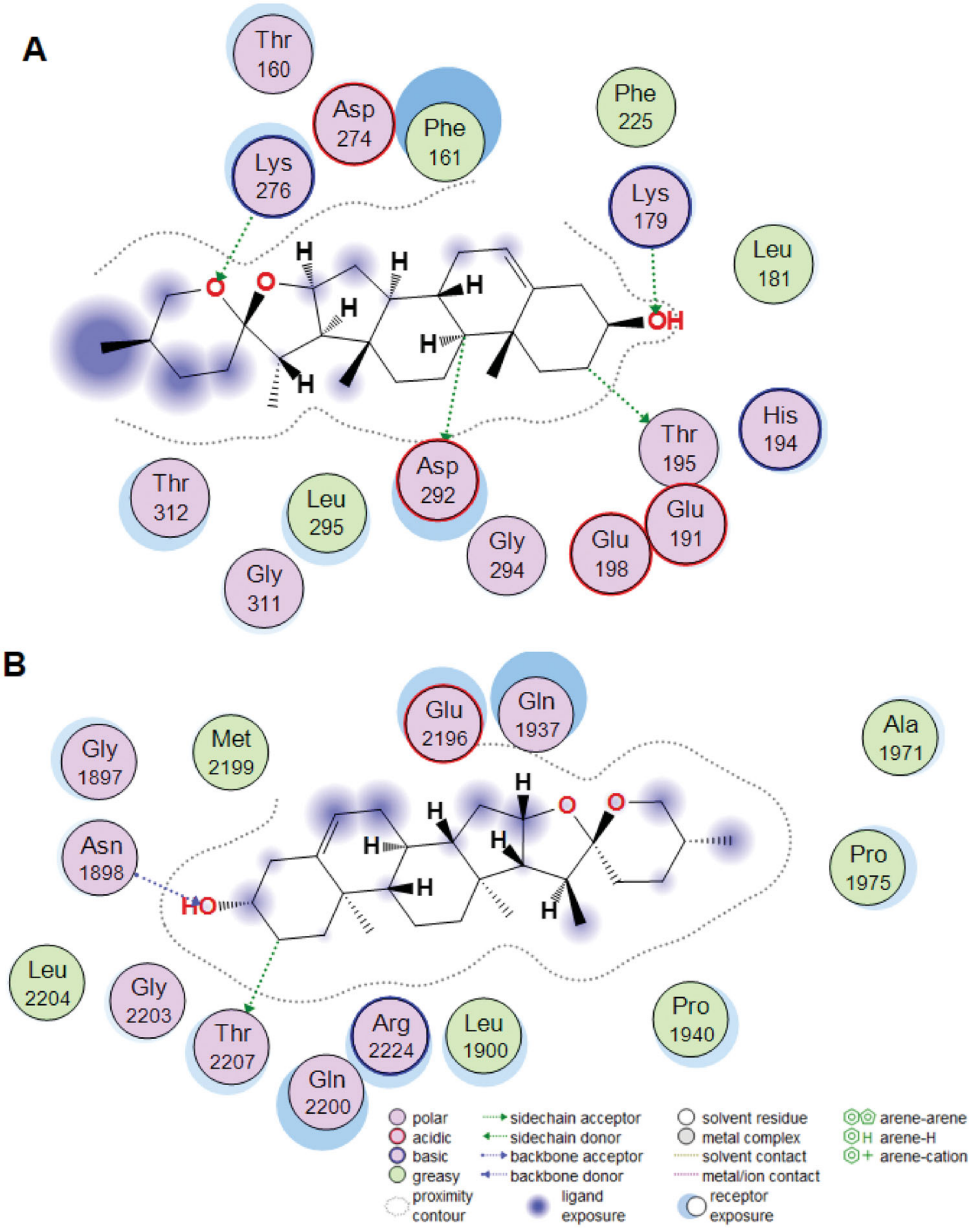
The interaction of the diosgenin (ligand) with targets of RP compounds, AKT1 (A) and mTOR (B), within their binding pockets.

As shown in Figure 8(A) the results of the additional analysis show that AKT1 compound forms multiple hydrogen bonds with the amino acids at the active site of the protein (LYS-276, LYS-179, THR-195, ASP-292), which can effectively bind small diosgenin molecule, allowing the compound to form a stable complex with it. In addition, the compound has high hydrophobicity and has strong hydrophobic interaction with surrounding hydrophobic amino acids (Thr-160, Phe-161, Leu-181, Glu-191, His-194, Glu-198, Phe-225, Gly-294, Leu-295, Asp-274, Gly-311, and Thr-319), which indicates that the compound matches the protein’s active pocket in a high degree and that their bonding is stable (Figure 8(A)). Additionally, the mTOR compound matches well with the diosgenin molecule as well. The binding sites include 14 amino acid residues with Ala-1971, Pro-1975, Gly-2203, and Met-2199 being added to the previously recorded 10 residues. There the contact sites of the positive compound are more consistent. The hydroxyl group at one end of the compound can form a hydrogen bond with the Asn-1898; Thr-2207 can form a weak hydrogen bond interaction with the hydrogen atom of the six-membered ring of the mTOR compound. In addition, the mTOR has a certain degree of hydrophobicity and has a strong hydrophobic interaction with the surrounding amino acids (Ala-1971, Met-2199, Leu-2204, Pro-1975, etc.) (Figure 8(B)). These strong interaction forces can effectively improve the stability of the diosgenin compound in the active pocket of the mTOR protein increasing the degree of matching with the protein.

Overall, the obtained results of the paper indicate that the RP compounds can be effectively used to target the kinase signaling pathways regulating cell growth. The thorough examination of the RP compounds using network pharmacology and molecular docking demonstrated that one of its ingredients—diosgenin can fit well into the binding pocket of important target proteins, AKT1 and mTOR, which play a critical role in the cellular proliferation, survival, and apoptosis processes. This means that diosgenin is a good candidate for further experimental validation to design a potent drug that could be used against various diseases including cancer that uses kinase signaling, specifically, the PI3K-AKT-mTOR pathway.

For AKT1 docking, the PDB structure of 4EKL was used which is bound with a highly selective pan-Akt inhibitor, GDC0068 by amino acids of Phe-22, Lys-40, Ala-91, Glu-95, Asp-53 (https://www.rcsb.org/sequence/4EKL) (Lin et al., 2012). For the mTOR structure, PDB 4JSV was used as a reference structure, which has been shown binding the ligand in a complex structure (Yang et al., 2013). Both above reported binding patterns of ligand binding to targets (Lin et al., 2012; Yang et al., 2013) are different from our modeling of diosgenin (ligand) binding to the targets of RP compounds, AKT1, and mTOR, within their binding pockets. However, the binding affinity showed a comparable in the contrast to LY294002 and Rapamycin. Thus the potent diosgenin binding to mTOR or AKT needs to be further investigated by dynamics both *in vitro* and *in vivo*.

The results suggest that diosgenin can interfere with kinase pathways which reregulate cell proliferation. This claim is confirmed by other reports (Shishodia & Aggarwal, 2006; Chiang et al., 2007; Srinivasan et al., 2009). Diosgenin is a natural product of steroidal sapogenin found in plants that inhibits cellular proliferation and induces tumor cell apoptosis through regulation of mTOR, AKT, JNK kinases signaling but direct binding and dephosphorylation are unknown (Shishodia & Aggarwal, 2006). It has been shown in breast cancer cells that diosgenin inhibits AKT activity which is an evidence of diosgenin inhibiting kinase especially AKT or mTOR (Chiang et al., 2007; Srinivasan et al., 2009). Thus our data is consistent with publications and their original data supported our findings. These imply that the RP’s ingredient, diosgenin, is a good candidate to be analyzed further.

Finally, to investigate whether we can apply the RP as a drug delivery machinery for kinase inhibitors with potent phosphatase activity to enhance the kinase inhibition, we tested the effect of the combination of RP with EGFR inhibitor (EGFRi) on phosphatase herbzyme activity. First, we tested the binding of RP with EGFR inhibitor Erlotinib Hydrochloride by fluorescence quenching analysis. As shown in Supplementary Figure 1 by fluorescence quenching compared to EGFRi or by wavelength peak shift compared to RP, the association between RP and EGFRi could be detected at excitation of 480 nm. Moreover, the binding did not show a significant effect on herbzyme phosphatase activity even though there is a slight increase due to the measurement sensitivity of the technical method of optical density analysis we usually use.

In summary, using network pharmacology we may identify the pathways that drug delivery carriers induced signaling and potent enzyme activity. The bioinformatics tool can provide an efficient approach to expedite the biochemical analysis to target the potent signaling and applying the drug delivery platform especially at nanoscale for enhanced efficiency (Liu et al., 2021), by the dual function of interface effect and herbzyme effect.

RP contains a lot of natural compounds which may be used for drug delivery as natural product delivery has been technically effective with proper medium (Zhang et al., 2015). For example, one of the compounds of RP is polysaccharides which may be applied for carriers by conjugating targeted drugs, such as kinase inhibitors for cancer treatment (Chourasia & Jain, 2004). This would be promising in further testing the conjugated RP-Kinase inhibitor complex in drug delivery of targeted kinase inhibitor drugs, with the natural product of RP, and phosphatase activity at nano-sized RP to enhance the kinase inhibitor efficancy *in vivo*.

## Conclusion

RP extract may against kinases through multiple pathways but one of the main signaling is AKT/mTOR. Network pharmacology and docking showed the consistent mTOR pathways targeted by RP. Thus, RP extract might be potentially used as a drug delivery carrier presented by a complex of the natural compound with kinase inhibitors to target kinases with intrinsic phosphatase herbzyme activity. Different from other types of drug delivery systems with potent combined kinase inhibitors, the RP extract compound may also exert a herbzyme of phosphatase function to target kinases. Thus the dual role of RP nano-extract shows the greatest potential of drug delivery by natural products complex.

## Note

1 Heatmap was plotted by http://www.bioinformatics.com.cn, an online platform for data analysis and visualization.
